# Objective quantification of posterior capsule opacification after cataract surgery with swept-source optical coherence tomography

**DOI:** 10.1186/s12886-023-03064-3

**Published:** 2023-07-05

**Authors:** Yu Zhou, Jing Xiang, Fang Xu, Ziyuan Jiang, Fang Liu

**Affiliations:** 1 Department of Ophthalmology, Tenth People’s Hospital Affiliated of Tongji University, Shanghai, China; 2 Department of Ophthalmology, Rui Jin Hospital, LuWan Branch, Shanghai Jiao Tong University School of Medicine, Shanghai, China

**Keywords:** Posterior capsule opacification, Swept-source optical coherence tomography, Posterior capsule thickness, Adaptive threshold algorithm

## Abstract

**Purpose:**

To evaluate the application of swept-source optical coherence tomography (SS-OCT) and pentacam scheimpflug tomography in posterior capsule opacification (PCO) severity assessment.

**Methods:**

The posterior capsule image region segmentation and adaptive threshold algorithm are used to process the SS-OCT scanned image to obtain the posterior capsule thickness (PCT). Scheimpflug tomography reconstructed and analysized by image J software can obtain the average gray value and evaluate the effectiveness with the two methods.

**Result:**

One hundred sixty-two IOL eyes of 101 patients were divided into two groups, laser group (65 eyes) with the mean PCT was 8.0 ± 2.7 pixel unit and the mean gray value of the eyes was 66 ± 33 pixel unit. However, these figures in the control group (97 eyes) were 5.0 ± 0.9 and 11 ± 17. The sensitivity, specificity and area under curve(AUC) of SS-OCT PCT were 85%, 74% and 0.942,the sensitivity, specificity and AUC of Pentacam gray value were 91%, 76% and 0.947, respectively. After using the multivariable model of generalized estimation equation to corrected the dependence of subjects' eyes, it was found that SS-OCT PCT, Pentacam gray value, low vision quality of life questionnaire (LVQ questionnaire) for distance vision, and mobility and lighting dimension were significantly correlated with the PCO score (*P* = 0.012, *P* = 0.001, *P* = 0.005, respectively).

**Conclusion:**

The region segmentation and adaptive threshold algorithm of posterior capsule image will accurately quantify the posterior capsule. Computer aided quantifications of posterior capsule are of great significance in the early surgical decision-making of PCO. The average occurrence time of most PCO was around 34 months, and the severity of PCO worsened with increasing postoperative time.

## Background

Posterior capsule opacification (PCO) is a commonly observed complication following cataract surgery, and is the primary cause of reduced visual acuity after the procedure. The fundamental cause of PCO is mesenchymal transdifferentiation, proliferation and migration of residual lens equatorial epithelial cells to the posterior capsule. Despite the removal of lens nucleus and cortex through phacoemulsification, the equatorial epithelial cells cannot be entirely eradicated. Consequently, these residual epithelial cells will undergo mesenchymal transdifferentiation, resulting in morphological changes, enhanced motor ability and migration of epithelial cells [1]. This process is accompanied by the deposition of extracellular matrix proteins (such as collagen and fibronectin) that negatively affect visual function [2]. The incidence of PCO is 10.65% ~ 95.68% [3]. There are two types of lens capsule opacification, anterior capsule opacification (ACO) and PCO [4], with no recognized treatment for ACO. Nd: YAG laser capsulotomy is a common treatment for PCO [5], which can effectively improve vision. However, it may lead to a series of complications, including IOL injury / dent, increased intraocular pressure (IOP), cystoid macular edema, retinal detachment and refractive changes [6–8]. To date, the degree of PCO is mainly subjectively or semi-quantitatively evaluated [9].

Currently, the degree of PCO is roughly judged by using slit lamp to visually measure posterior capsule in pupil area. This method is subjective and relies heavily on the examiner's clinical experience, leading to potential bias. Additionally, it does not provide accurate information regarding the range, location, thickness, and density of the opacification. Pentacam based on the principle of Scheimpflug fissure photography has been used in anterior segment examination. While the Pentacam imaging method, which produces three-dimensional images of the anterior segment and clearly displays the degree of PCO, has been developed [10], it is not widely used in clinical settings due to its high cost and specialized equipment. On the other hand, the IOLMaster 700, primarily used for measuring intraocular lenses (IOLs), is noninvasive, repeatable, and widely available. It can also provide a Swept-source optical coherence tomography (SS-OCT) scanning, which can display detailed information of the PCO through cross-sectional images but lacks quantitative measurements. Therefore, an objective and effective method is necessary to assess PCO morphology. In this study, we aim to compare the Pentacam anterior segment analysis system and IOLMaster 700 to identify such a method.

## Patient and methods

### Patient

This cross-sectional study recruited patients after cataract surgery in Tenth People's hospital of Tongji University. This study was approved by the Clinical Research Ethical Committee of the Shanghai Tenth People’s Hospital affiliated with Tongji University and adhered to the principles of the Declaration of Helsinki (clinical study registered at www.chictr.org.cn, accession number ChiCTR2100043179). The data of 162 eyes of 101 patients who underwent cataract surgery in Tenth People's Hospital of Tongji University from April 2020 to November 2020 were collected. The age ranged from 18 to 88 years, with an average of (67.78 ± 9.94) years. The inclusion criteria were: ①Intraocular lens was implanted without complications; ②The posterior capsule was intact, and there was no obvious deviation of IOL position by slit lamp microscope; ③Normal pupil activity; ④Intraocular pressure (IOP) is between 10 and 20 mmHg, and can cooperate to complete the examination and follow-up. Patients with corneal related diseases, retinal detachment after silicone oil injection, glaucoma, uveitis, eye trauma, complex ophthalmic surgery history and serious systemic diseases were excluded.

### Method

**Fig. 1:**
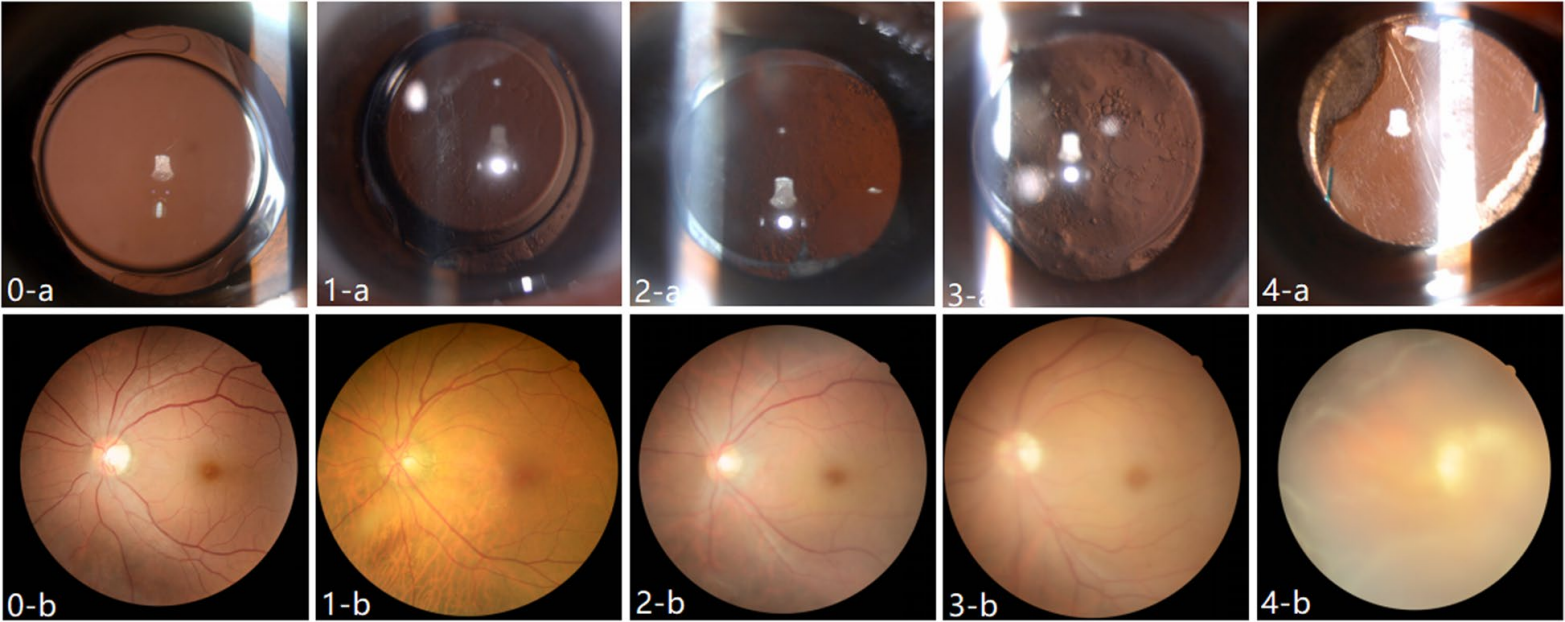
**a** PCO slit lamp retroillumination images, **b** fundus image, PCO score was graded as follows: 0 = transparent,no opacity or opacity limited to the peripheral capsule; 1 = any wrinkling or opacity of the capsule affecting a circle 4 mm in diameter and centred on the visual axis, but not compromising the view of the posterior pole; 2 = central/paracentral opacity as described above sufficient to degrade details of the macula slightly, but still allowing the cup/disc ratio to be readily ascertained; 3 = central/paracentral opacity as defined above, but sufficient to make ascertainment of the cup/disc ratio difficult; 4 = central/paracentral opacity as defined above, but sufficient to make visualisation of fundus details difficult

All patients underwent routine preoperative ophthalmic examination, including best corrected visual acuity (BCVA) (LogMAR visual acuity), measurement of intraocular pressure with tx.20 non-contact tonometer (Canon, Japan), full mydriasis with compound tropicamide eye drops (medori), slit lamp digital camera (Topcon SL-D701, Japan) and fundus red light reflex photography. + 90 D be used to check vitreous and fundus. Optical coherence tomography (OCT) (Zeiss Cirrus HD OCT 5000,Germany) is performed to further determine whether there is macular disease. 162 eyes were divided into laser group(*n* = 65) and control group(*n* = 97) according to slit lamp retroillumination image and visual acuity by two experienced observers, who also subjectively scored the PCO using slit lamp image with following Fig. 1 [11]. When the two observers differ, the decision was made by another chief ophthalmologist.

The Pentacam (OCULUS Pentacam AXL,Germany) uses the automatic examination mode (50 frames / 2S) to obtain the Scheimpflug slit lamp photos of the patient's posterior capsule. Select the IOL plane image on the coronal plane, that is, the Pentacam image of the posterior capsule, and then use Image J software to analyze its pixels to obtain the average pixel gray value within the diameter of 4 mm and 3 mm in the center.

Swept-source OCT of the posterior capsule was performed using the IOLMaster 700 (Zeiss IOLMaster 700, Germany) device, the patient takes the sitting position, puts his head squarely on the mandibular support, aligns the affected eye with the lens, instructs him to use the affected eye to look at the internal fixation point and try not to blink, and uses the operating handle to find the best signal-to-noise ratio to take images and store them.

Each patient fills in the quality of life table. The quality of life can be objectively measured by low vision quality-of-life questionnaire (LVQ questionnaire) evaluation questionnaire in the proportion of 0–125, where 0 indicates the extremely poor quality of life and 125 indicates the best quality of life that can be achieved. LVQ questionnaire is divided into four dimensions: distance vision, mobility and lighting, adjustment, reading and fine work and activities of daily life.

### Calculation algorithm of average pixel gray value of posterior capsule Pentacam image

**Fig. 2:**
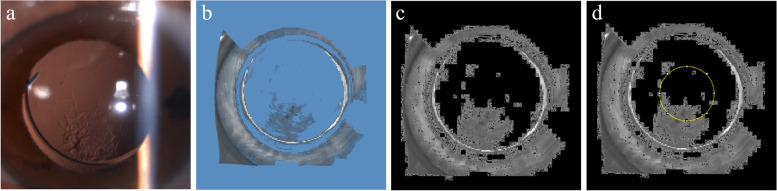
Scheimpflug image processing steps. **a** slit lamp retroillumination images (**b**) Pentacam original image; (**c**) set the gray value of the background color to 0;(d)Use Image J software to select the area within 3 mm diameter (small white circle)

After obtaining the Pentacam image of the posterior capsule, first intercept the target part in the image, second it needs to be processed and calculated within 3 mm diameter of the center. The specific algorithm is as follows:

1. Firstly, the color image is converted into a gray image, that is, the pixel value range of each pixel is 0–255.
2. Calculate the gray value of the background color in the figure (blue in the figure), then set the gray value of the background color to 0 (i.e. pure black), and the gray values of other colors remain unchanged.
3. Put the gray processed into Image J software, select the area within 3 mm diameter in the center, and then calculate the average gray value in the area. The processed picture is shown in Fig. 2.

### Calculation algorithm of average posterior capsule thickness of SS-OCT image

The main challenges of accurate segmentation of posterior capsule are detailed as follows:

- The anatomical structure of posterior capsule is very weak, especially in patients with transparent posterior capsule, it is difficult to extract the region of interest.
- The noise of SS-OCT will make some sporadic white spots in the image, which will affect the results.

**Fig. 3:**
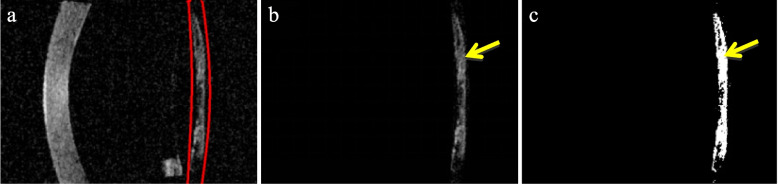
SS-OCT image processing steps. **a** determine the IOL-posterior capsular space (the two red lines). **b** Preserve target area image (yellow arrow). **c **The threshold adaptive algorithm is used to further reduce the influence of noise, binarize the image (yellow arrow)

**Fig. 4:**
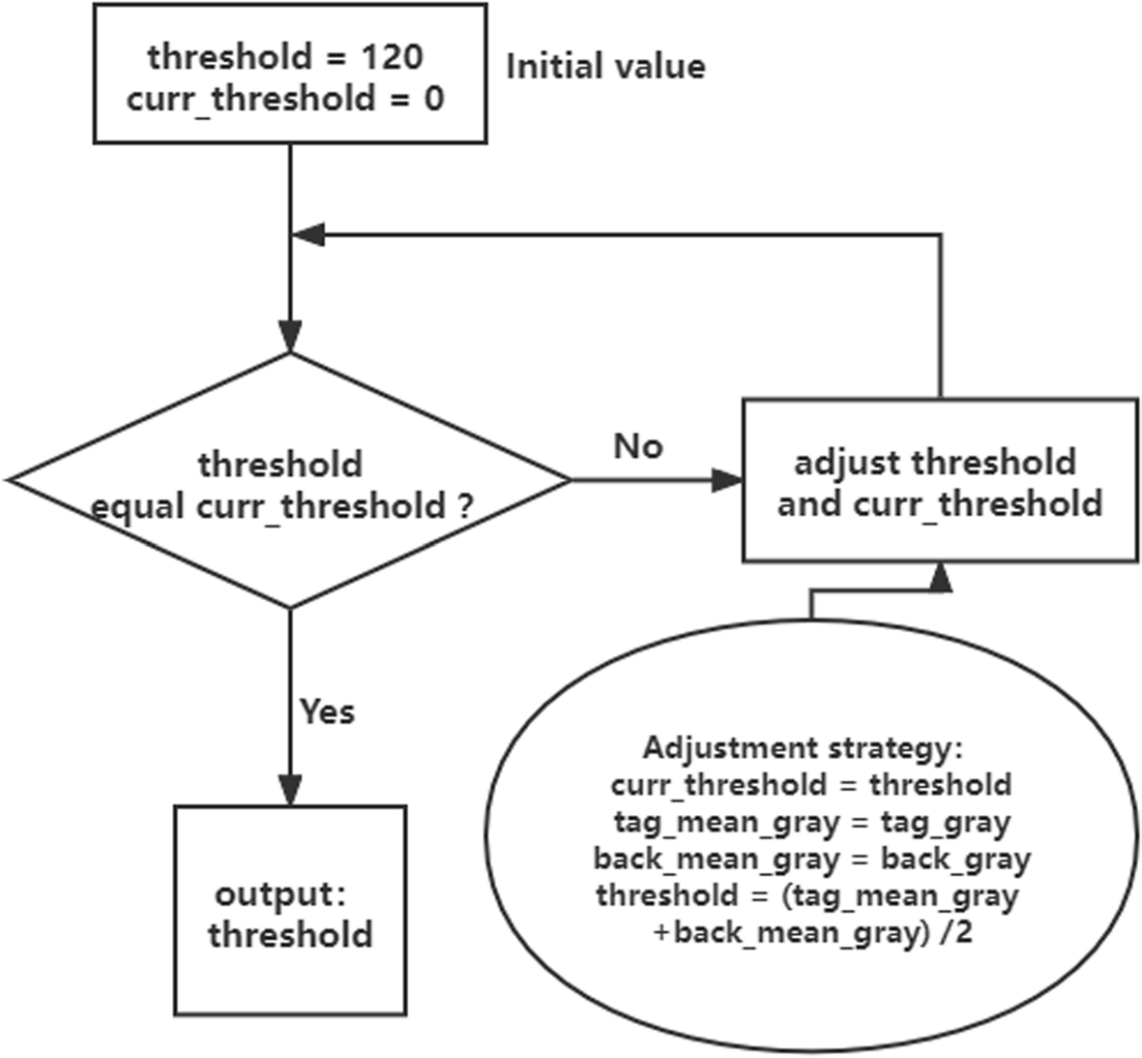
Pseudo code of threshold adaptive algorithm

Considering the current challenges in the field of posterior capsule segmentation, this paper proposes a method to calculate the average thickness of posterior capsule based on SS-OCT image, it is mainly divided into the following parts:

1. In order to avoid the influence of noise on the picture, accurately select the part of interest in SS-OCT image. As shown in Fig. 3a, the area in the middle of the red line is the required target area.
2. Using the image region of processing algorithm, the image of the target region is retained, and all pixels in other regions are set to (0,0,0). The image processing is completed as shown in Fig. 3b.
3. The processed image is converted into a gray image.
4. The gray image is binarized according to the set threshold, that is, the pixel value is set to 0 or 255 (the image has only white and black). It is specified that the pixels representing the posterior capsule are white and the background is black.
5. As can be seen from Fig. 3b, the image after selecting the target area effectively reduces the impact of machine noise. At this time, the threshold adaptive algorithm is used to further reduce the impact of noise, and finally the average thickness of posterior capsule (in pixel units) is obtained (Fig. 3c). The steps of threshold adaptive algorithm are shown in Fig. 4.

## Statistical analysis

All data were statistically analyzed by spss 21.0. Results are expressed as means ± standard deviations with ranges. Chi-square test and Mann Whitney U test were used for comparison between the laser group and the control group, the correlation between various parameters and postoperative time interval was analyzed by Spearman's correlation test. For all analysis at the eye level (thickness and gray scale), the generalized estimation equation with exchangeable correlation matrix was used to correct the intra ocular dependence of subjects, and it was adjusted for age, sex, education and the number of months after surgery. The agreement between interobservers was measured using the kappa coefficient. The ROC curve, specificity and sensitivity curve of Pentacam gray scale and IOLMaster 700 thickness value were drawn respectively, and the area under the curve (AUC) was processed by computer. AUC = 1.0 is the most ideal detection index, and AUC < 0.5 indicates no diagnostic value.

## Result

The study evaluated 162 eyes of 101 patients. A total of 162 IOLs were implanted, of which: 54 (33.3%) ZCB00 (Johnson & Johnson Vision, Santa Ana, CA, USA), 39 (24.1%) ZA9003 (Johnson & Johnson Vision, Santa Ana, CA, USA), 25 (15.4%) Akreos Adapt AO (Bausch & Lomb; Rochester, NY, USA), 23 (14.2%) Aspira-aAY (HumanOptics AG, Erlangen, Germany) and 21 (13.0%) AR40e (AMO, Santa Ana, CA, USA). And including 65 cases in the laser group and 97 cases in the control group. The age of patients in the laser group was 66 ± 13 years(range from 19 to 89 years); The age of patients in the control group was 69 ± 7 years(range from 47 to 87 years).

The Laser group and Control group’s baseline characteristics and difference was shown in Table 1. Sex and Cylinder did not significantly affect Laser group and Control group (Mann–Whitney U test, all *P* > 0.05).The mean BCVA in laser group was 0.06 ± 0.10(range from 0 to 0.52),The mean BCVA in the control group was 0.47 ± 0.48(range from 0 to 2), with significant statistical differences (*P* < 0.001). The mean LVQ questionnaire score in laser group was 56.94 ± 29.07(range from 3 to 114),The mean LVQ questionnaire score in the control group was 92.59 ± 18.82(range from 44 to 118), with significant statistical differences(*P* < 0.001).The mean PCT in laser group was 8 ± 2.70(range from 5.40 to 20.5), The mean PCT in the control group was 5 ± 0.90(range from 3.10 to 7.10), with significant statistical differences (*P* < 0.001). Within 3 mm diameter, the mean gray value of the eyes in the laser group was 66 ± 33 (range from 6 to 176); The average gray value of the eyes in the control group was 11 ± 17 (range from 0 to 91), with significant statistical differences (*P* < 0.001). Kappa coefficient between two observers across the subjective PCO score was good (0.94, 95% CI 0.90–0.98).

**Table 1:** Baseline characteristics and difference between Laser group and Control group

Parameter	Laser group (*n* = 65)	Control group (*n* = 97)	*P*
Age (yrs)	66 ± 13	69 ± 7	0.415
Sphere (D)	-1.84 ± 1.60	-1.05 ± 1.50	0.001
Cylinder (D)	-1.21 ± 1.12	-0.91 ± 0.69	0.266
IOL type, N(%)			0.013
ZCB00	15 (23.1%)	39 (40.2%)	
ZA9003	20 (30.8%)	19 (19.6%)	
AR40E	14 (21.5%)	7 (7.2%)	
Akreos AO	9 (13.8%)	16 (16.5%)	
Aspira-aAY	7 (10.8%)	16 (16.5%)	
LVQ questionnaire	56.94 ± 29.07	92.59 ± 18.82	0.000
Distance vision, mobility and lighting	28.66 ± 14.21	44.92 ± 9.28	0.000
Adjustment	8.17 ± 3.70	12.61 ± 2.66	0.000
Reading and fine work	9.58 ± 6.09	17.71 ± 5.51	0.000
Activity of Daily living	10.52 ± 6.57	17.24 ± 3.60	0.000
BCVA(logMAR)	0.06 ± 0.10	0.47 ± 0.48	0.000
Postoperative time interval (months)	23.89 ± 20.13	42.46 ± 22.89	0.000
Gray value (pixel unit)	66 ± 33	11 ± 17	0.000
PCT (pixel unit)	8 ± 2.7	5 ± 0.90	0.000

*IOL* Intraocular Lens, *LVQ questionnaire* low vision quality-of-life questionnaire, *BCVA* Best-corrected visual acuity, *logMAR* logarithm of the minimum angle of resolution, *PCT* posterior capsule thickness. Continuous variables are represented as mean value ± standard deviation, and the categorical variables are showed as frequencies. Chi-square test and Mann–Whitney U test statistically significant at *p* < 0.05

The Table 2 presents the mean values with standard deviation of postoperative time intervals for different PCO scores. Specifically, when the PCO score was 0, 1, 2, 3, and 4, the corresponding mean postoperative times were 14.06 (± 13.81), 34.51 (± 23.70), 37.61 (± 22.68), 40.5 (± 23.19), and 47.68 (± 23.88) months, respectively. Furthermore, the results of the Spearman's correlation test indicated a significant positive correlation between postoperative time interval and the PCO score (*r* = 0.530, *P* < 0.01), Pentacam gray value (*r* = 0.707, *P* < 0.01) and SS-OCT PCT (*r* = 0.631, *P* < 0.01).

**Table 2:** Mean values with standard deviation of various parameters for different PCO scores

	PCO score				
	0	1	2	3	4
PCO score, N (%)	58 (35.8%)	41 (25.3%)	28 (17.3%)	16 (9.9%)	19 (11.7%)
Postoperative time interval (months)	14.06 ± 13.81	34.51 ± 23.70	37.61 ± 22.68	40.5 ± 23.19	47.68 ± 23.88
Gray value (pixel unit)	5.74 ± 10.28	31.21 ± 29.28	51.04 ± 35.44	50.74 ± 25.26	81.24 ± 41.14
PCT (pixel unit)	4.94 ± 0.95	5.79 ± 1.32	6.68 ± 1.74	7.30 ± 1.53	9.52 ± 4.13

*PCO* posterior capsule opacification. Continuous variables are represented as mean value ± standard deviation, and the categorical variables are showed as frequencies

From the multivariable model of generalized estimation equation (Table 3), we corrected age, gender, education, the number of months after surgery and the dependence of subjects' eyes. It was found that SS-OCT PCT, Pentacam gray value, LVQ QUESTIONNAIRE Distance vision, Mobility and Lighting dimension were significantly correlated with the PCO score (*P* = 0.012, *P* = 0.001, *P* = 0.005, respectively).

**Table 3:** Associations Between objective measurements and PCO score

	PCO Score					
	Unadjused			Adjusted		
	β	Exp(β)_u_	*P* value	β	Exp(β)_a_(95%CI)	*P* value
Eye ID	-0.022	0.978	0.925			
Gray value	0.048	1.050	0.000	0.02	1.02(1.01,1.04)	0.001
PCT	1.029	2.799	0.000	0.33	1.39(1.07,1.80)	0.012
BCVA(logMAR)	-4.972	0.007	0.000	0.06	1.07(0.15,7.62)	0.950
LVQ questionnaire	-0.072	0.930	0.000			
Distance vision, Mobility and Lighting	-0.158	0.854	0.000	-0.10	0.91(0.85,0.97)	0.005
Adjustment	-0.401	0.670	0.000	0.07	1.07(0.89,1.29)	0.461
Reading and fine work	-0.202	0.817	0.000	0.01	1.01(0.92,1.11)	0.844
Activity of Daily living	-0.322	0.725	0.000	-0.02	0.98(0.87,1.12)	0.789

*Eye ID* right/left eye, *LVQ questionnaire* low vision quality-of-life questionnaire, *BCVA* Best-corrected visual acuity, *logMAR* logarithm of the minimum angle of resolution, *PCT* posterior capsule thickness. β = coefficient value. generalized estimation equation was used to account for the intereye correlation, each variant assessed in a model, controlling for age, sex, education and the number of months after surgery. statistically significant at *p* < 0.05

**Fig. 5:**
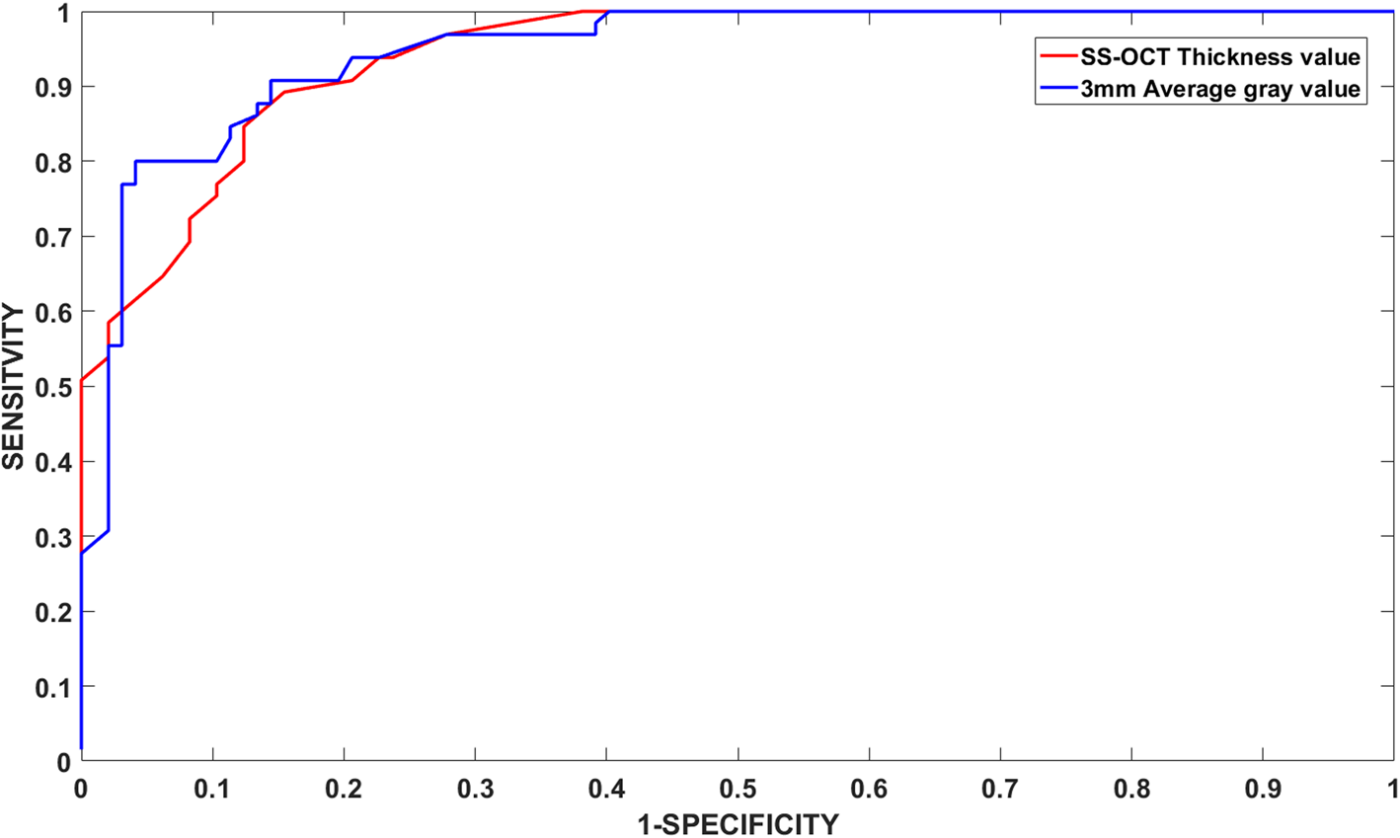
ROC curve of 3 mm gray value (blue) and posterior capsule thickening (red)

The receiver operating characteristic (ROC) curves of the 3 mm average gray value measured with the Scheimpflug device (blue), the posterior capsule thickening measured with the swept-source optical coherence tomographer(red). The area under the ROC was 0.947 and 0.942, respectively. AUC values are more than 0.5 and shown that both devices have application value (Fig. 5).

**Fig. 6:**
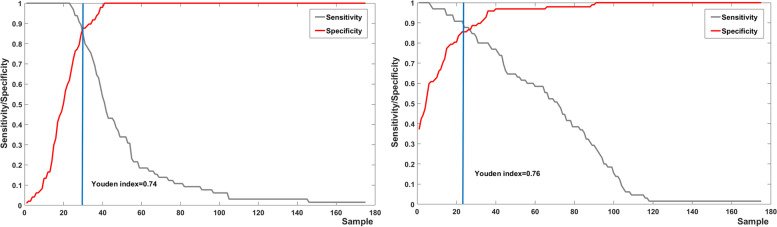
Specificity and sensitivity curve of PCT (left) and average gray value of 3 mm area in Pentacam picture(right)

The specificity and sensitivity curves of the average thickness of posterior capsule in IOL master image and the average gray value in 3 mm area of posterior capsule Pentacam image are shown in Fig. 6, the sensitivity and specificity of SS-OCT PCT were 85% and 74%, the sensitivity and specificity of Pentacam gray value were 91% and 76%, respectively. It can be seen that the Youdan indexes of the two are 0.74 and 0.76 respectively. Two methods are used to determine whether laser is needed and the results are the same, both methods are effective (Youden index ≥ 0.5).

## Discussion

PCO is a common long-term complication of cataract phacoemulsification. Multiple studies have investigated various strategies to prevent and treat PCO, including surgical techniques, drug research [12] and intraocular lens design [13, 14]. Objective quantitative measurement of PCO is crucial in evaluating the effectiveness of such interventions and assessing the clinical course. Although several imaging systems have been reported, there remains no consensus on the optimal quantitative method for PCO analysis. Prior efforts to design an objective system such as the Posterior Capsule Opacification (POCO) [15], Automated Quantification of After-Cataract [16], and the AA System [17], have relied on analyzing slit lamp retroillumination images. However, these systems are partially subjective and limited by reflection artifacts or Purkinje spots.

Previous study has indicated that Pentacam rotating Scheimpflug system has high repeatability for quantitative PCO, which has good correlation with the results obtained by using POCOman system to analyze the retroillumination photos based on slit lamp [18]. However, the deficiency of previous studies is that the existance of the background color (blue) of Scheimpflug tomography image may lead to the deviation of results. In our study, we remove the background color and objectively quantify the gray value of the posterior capsule. As depicted in Fig. 2, the visualization of posterior capsule opacity morphology became more intuitive upon removal of the background color. A critical issue in gray image analysis is to define the region of interest (ROI). We choose the 3.0 mm area in the center of IOL as the objective measurement range to avoid the occlusion of the anterior capsule to IOL. We find that this method has a great sensitivity (91%) in detecting whether posterior capsular opacity needs laser treatment. According to Youden index analysis, the gray value of 22 pixel units or above strongly indicates that the posterior capsule opacity reaches the degree of laser treatment. This method has many advantages: 1.there is no flash reflection in the sectional image; 2.the generation of tomographic images is almost independent of the operator when Pentacam automatic release mode is used; 3. The speed of acquiring images is faster; 4. the operators are not required to master complex professional knowledge. Operator dependent is limited to selecting the ROI to limit the size of the edge detection range. Nonetheless, a notable contrast in the scattering light density measured by Scheimpflug imaging system is evident among intraocular lenses fashioned from different optical materials. Additionally, the Scheimpflug videophotography system is unable to distinguish slit image signals originating from the posterior capsule from those of the posterior IOL surface [19]. As a result, the methodology employed by the Scheimpflug imaging system is restricted in the assessment of the degree of posterior capsule opacity after implantation of intraocular lenses made of different optical materials.

Previous study demonstrated that posterior capsule thickness (PCT) is the distance between two reflectivity peaks with IOL reflectivity. The peak intensity is the maximum height of the last peak of PCO. Measurements were made at only three points, the central point, temporal point and nasal point [20]. Due to the Irregular distribution of PCO, this method has limitations. Our research takes the lead in using the average pixel unit thickness to measure PCT, breaking through the limitations of previous methods and making the PCT value more accurate. A major challenge of this technology is the noise formed by B-scan tomography of SS-OCT. In order to reduce the influence of artifacts, we introduce an adaptive algorithm to calculate the threshold, and then eliminate the artifacts as much as possible to improve the accuracy. It has been previously reported that visual acuity is significantly correlated with posterior capsule thickening (PCT). According to the ROC curve analysis, the AUC of PCT is 0.942, suggesting that PCT has high sensitivity and accuracy in the diagnosis of PCO. PCT is 5.86 pixel units or above, suggesting that further treatment should be carried out.

PCO causes light scattering in the visual axis and visual impairment [21], PCO also reduces all aspects of visual function, including contrast sensitivity, glare, Color vision and stereo vision [22–24]. In the early stage of PCO, due to the uneven posterior capsule turbidity, the light can still pass through the turbid gap, and the visual impairment is not obvious, but the light scattering is significantly increased, and the visual function is affected. This is also related to the turbidity type of after-cataract, the involvement of visual axis area, the density of turbidity, etc. [25, 26]. Previous studies have shown that the relationship between VA and the severity of PCO is nonlinear [23], and BCVA cannot fully reflect the degree of posterior capsule involvement, which is consistent with our study (*P* = 0.91). Our study showed that the LVQ questionnaire’s Distance vision, Mobility and Lighting dimension (*P* = 0.01) seems to be more appropriate than VA in assessing visual function, so as to understand the subjective symptoms of PCO patients and guide the timing of laser intervention. Furthermore, our findings revealed a substantial positive correlation between the severity of PCO and the postoperative time interval. Additionally, the correlation between the postoperative time interval and Pentacam gray value, as well as SS-OCT PCT, was found to be greater than that observed for PCO score. These devices have the potential to detect subclinical PCO and may also aid in predicting the rate of PCO progression.

In conclusion, our study innovatively proposed the region segmentation and adaptive threshold algorithm of posterior capsule image, eliminated the influence of noise, accurately calculated the average thickness and average gray value of posterior capsule, which enabled the identification of subclinical changes and more comprehensively evaluated the severity of posterior capsule turbidity. SS-OCT PCT, Pentacam tomography gray value and LVQ questionnaire’s Distance vision, Mobility and Lighting dimension are of great significance in the early surgical decision-making of PCO. As indicators for the referral of Nd: YAG laser capsulotomy, its can distinguish early beneficial capsulotomy from early unhelpful capsulotomy, and provide better guidance for the situation where the risk–benefit ratio may not be clear.

## Data Availability

The datasets used and analyzed during the current study are available from the corresponding author on reasonable request.

## Abbreviations

PCO: Posterior capsule opacification
SS-OCT: Swept-source optical coherence tomography
PCT: Posterior capsule thickness
ROC: Receiver operating characteristic
AUC: Area under the curve
ROI: Region of interest
LVQ questionnaire: Low vision quality-of-life questionnaire
BCVA: Best-corrected visual acuity
logMAR: Logarithm of the minimum angle of resolution
